# 16S rRNA sequencing analysis of the oral and fecal microbiota in colorectal cancer positives versus colorectal cancer negatives in Iranian population

**DOI:** 10.1186/s13099-024-00604-0

**Published:** 2024-02-20

**Authors:** Sama Rezasoltani, Mehdi Azizmohammad Looha, Hamid Asadzadeh Aghdaei, Seyedesomayeh Jasemi, Leonardo Antonio Sechi, Maria Gazouli, Amir Sadeghi, Shirin Torkashvand, Reyhaneh Baniali, Hartmut Schlüter, Mohammad Reza Zali, Mohammad Mehdi Feizabadi

**Affiliations:** 1 Section Mass Spectrometric Proteomics, Diagnostic Center, University Medical Center Hamburg-Eppendorf (UKE), 20246 Hamburg, Germany; 2 Division of Oral Microbiology and Immunology, Department of Operative Dentistry, Periodontology and Preventive Dentistry, RWTH University Hospital, 52057 Aachen, Germany; 3 Basic and Molecular Epidemiology of Gastrointestinal Disorders Research Center, Research Institute for Gastroenterology and Liver Diseases, Shahid Beheshti University of Medical Sciences, Tehran, 19835-178 Iran; 4 Department of Biomedical Sciences, University of Sassari, Viale San Pietro 43b, 07100 Sassari, Italy; 5 Struttura Complessa Microbiologia e Virologia, Azienda Ospedaliera Universitaria, 07100 Sassari, Italy; 6 Department of Basic Medical Sciences, Laboratory of Biology, Medical School, National and Kapodistrian University of Athens, Athens, Greece; 7 Gastroenterology and Liver Diseases Research Center, Research Institute for Gastroenterology and Liver Diseases, Shahid Beheshti University of Medical Sciences, Tehran, 19835-178 Iran; 8 Department of Microbiology, School of Medicine, Tehran University of Medical Sciences, Tehran, 19835-178 Iran; 9 Thoracic Research Center, Imam Khomeini Hospital Complex, Tehran University of Medical Sciences, Tehran, Iran

**Keywords:** Colorectal cancer, Oral microbiota, Fecal microbiota, Early detection, 16S rRNA sequencing

## Abstract

**Background:**

Colorectal cancer (CRC) poses a significant healthcare challenge, accounting for nearly 6.1% of global cancer cases. Early detection, facilitated by population screening utilizing innovative biomarkers, is pivotal for mitigating CRC incidence. This study aims to scrutinize the fecal and salivary microbiomes of CRC-positive individuals (CPs) in comparison to CRC-negative counterparts (CNs) to enhance early CRC diagnosis through microbial biomarkers.

**Material and methods:**

A total of 80 oral and stool samples were collected from Taleghani Hospital, Shahid Beheshti University of Medical Sciences, Tehran, Iran, encompassing both CPs and CNs undergoing screening. Microbial profiling was conducted using 16S rRNA sequencing assays, employing the Nextera XT Index Kit on an Illumina NovaSeq platform.

**Results:**

Distinct microbial profiles were observed in saliva and stool samples of CPs, diverging significantly from those of CNs at various taxonomic levels, including phylum, family, and species. Saliva samples from CPs exhibited abundance of *Calothrix parietina*, *Granulicatella adiacens*, *Rothia dentocariosa*, and *Rothia mucilaginosa*, absent in CNs. Additionally, *Lachnospiraceae* and *Prevotellaceae* were markedly higher in CPs' feces, while the Fusobacteria phylum was significantly elevated in CPs' saliva. Conversely, the non-pathogenic bacterium *Akkermansia muciniphila* exhibited a significant decrease in CPs' fecal samples compared to CNs.

**Conclusion:**

Through meticulous selection of saliva and stool microbes based on Mean Decrease GINI values and employing logistic regression for saliva and support vector machine models for stool, we successfully developed a microbiota test with heightened sensitivity and specificity for early CRC detection.

## Background

Colorectal cancer (CRC) stands as a leading cause of cancer-related mortality in both developed and developing countries [1]. Implementation of population-based CRC screening has demonstrated a potential to reduce CRC incidence, garnering strong recommendations [3, 4]. Notably, over 85% of CRC cases originate from pre-malignant adenoma polyps, emphasizing the preventive nature of early detection [5]. The primary objective of CRC screening is to identify pre-symptomatic neoplastic lesions, thereby reducing the overall incidence through timely intervention and examination [6].

The prevailing CRC screening approaches involve fecal immunochemical tests (FIT) coupled with subsequent colonoscopies for positive cases, or periodic endoscopic procedures such as flexible sigmoidoscopy every 5 years or colonoscopy every 10 years [8, 9]. Ongoing considerations include alternative screening methods like fecal DNA analysis and CT colonography [5]. However, the efficacy of any screening program hinges on two pivotal factors: compliance and accuracy [10]. Despite the success observed in various strategies, overall individual compliance remains suboptimal, with rates falling below 52% in CRC screening initiatives [5]. Therefore, there is a growing consensus that novel strategies, encompassing the amalgamation of established tests or the introduction of convenient screening alternatives, could significantly enhance population-based CRC screening adherence [11, 12].

Remarkably, altered microbiota composition has emerged as a potential foundation for a highly sensitive and specific CRC screening test [13–18]. Beyond microbiota, their proteins and metabolites contribute to CRC pathogenesis, with reciprocal interactions influencing host proteins and metabolites in CRC development [19]. Significantly, signatures derived from the abundance of bacterial proteins, particularly those associated with signal transduction systems like sensory proteins, hold promise in distinguishing between healthy and diseased states [19].

In this context, our study represents a continuation of previous efforts focused on early CRC detection based on microbial biomarkers [15, 20, 21]. We aim to assess fecal and oral microbiota through 16S rRNA sequencing analysis, exploring the abundance and variation of pathogenic oral and fecal microbiota composition between CRC-positive individuals (CPs) and CRC-negative counterparts (CNs) in the Iranian population. Additionally, we investigate the status of nonpathogenic microorganisms, including probiotics and short-chain fatty acid (SCFA)-producing bacteria, in the feces of CPs compared to CNs. Ultimately, we endeavor to develop classifier models utilizing oral and fecal microbiota profiles, with the intent of enhancing the diagnostic capabilities for early CRC detection with high sensitivity and specificity.

## Results

### Demographic results

Demographic characterization of participants with related *p*-value between CPs and CNs are presented in Table 1. The population study was characterized by similar distributions of gender, viral infection, alcohol consumption and dietary habit. The profession, family history, disease and surgical history, smoking habit and physical activity had significant differences between the CPs and CNs based on *p*-value.

**Table 1:** Demographic characteristics of CRC positives (CPs) and CRC negatives (CNs)

Variables	Group		*p*-value
	CPs	CNs	
Age, mean (SD)	58.88 (15.18)	45.40 (13.04)	0.007
Gender, N (%)			0.512
Female	12 (48.00)	5 (33.33)	
Male	13 (52.00)	10 (66.67)	
Profession, N (%)			0.045
Butler	0 (0.00)	1 (13.33)	
Employee	5 (4.00)	8 (20.00)	
Housewife	13 (48.00)	4 (26.67)	
Retired	7 (24.00)	2 (0.00)	
Family history, N (%)			0.036
No	13 (52.00)	13 (86.67)	
Yes	12 (48.00)	2 (13.33)	
Disease history or surgical history, N (%)			0.018
No	11 (44.00)	12 (80.00)	
Yes	14 (56.00)	3 (20.00)	
Viral infection, N (%)			1.000
No	23 (92.00)	15 (100.00)	
Yes	2 (8.00)	0 (0.00)	
Smoking, N (%)			0.033
No	19 (76.00)	15 (100.00)	
Yes	6 (24.00)	0 (0.00)	
Alcohol consumption, N (%)			0.545
No	24 (96.00)	15 (100.00)	
Yes			
Nutrition diet, N (%)			0.225
All food	16 (64.00)	11 (73.33)	
High fruit and vegetable	2 (8.00)	3 (20.00)	
High meat consumption	7 (28.00)	1 (6.67)	
Physical activity, N (%)			0.001
No	23 (92.00)	6 (40.00)	
Yes	2 (8.00)	9 (60.00)	

The independent *t*-test was used to compare the mean of age between CRC and CRC negative. The Fisher exact test or exact Pearson Chi-Square was used to evaluate the relation between categorical variables and group

### 16S rRNA sequencing analysis of clinical samples:

#### Top 10 microbes with more abundance in CPs versus CNs

**Fig. 1:**
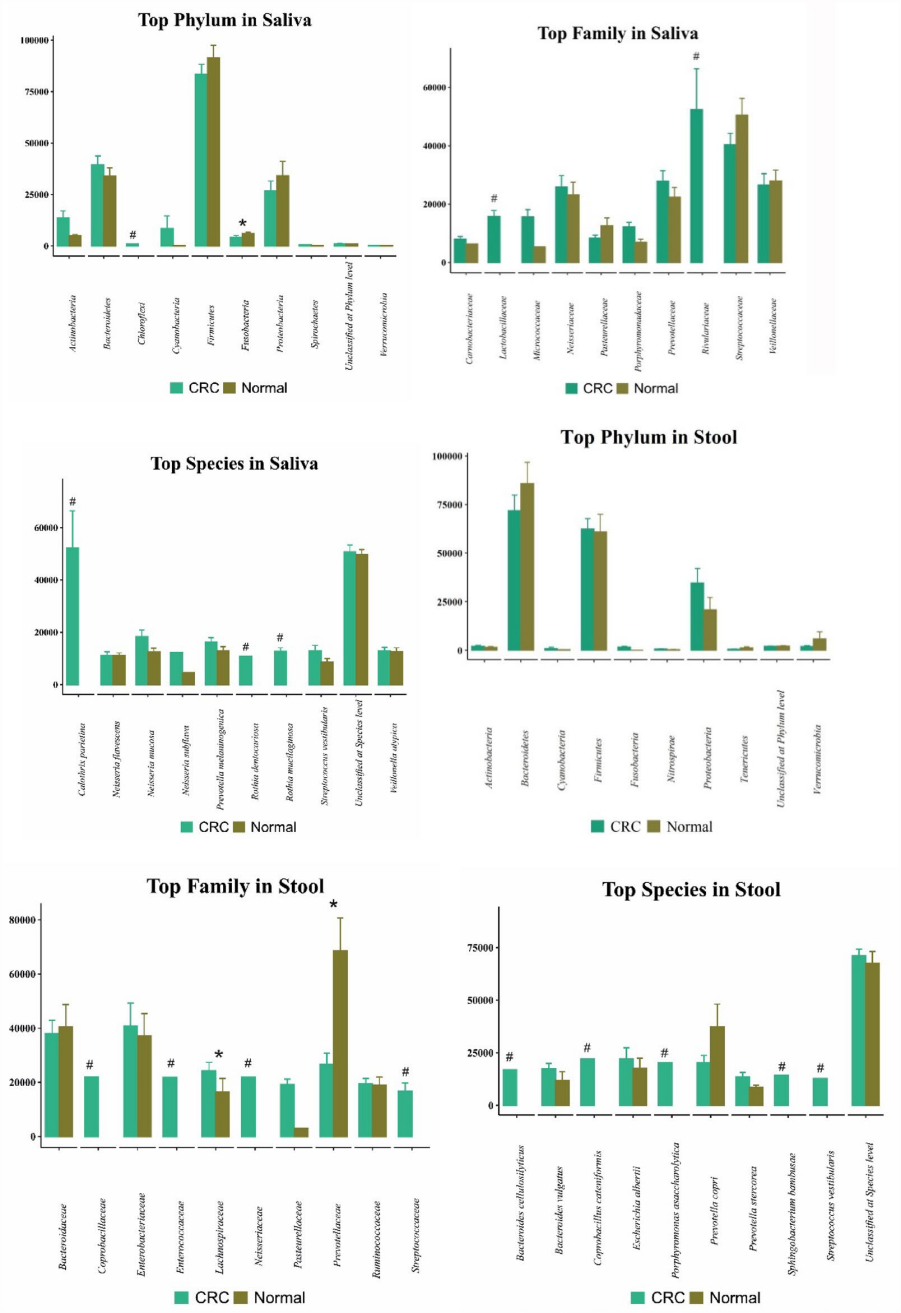
The frequency of top 10 bacteria that were most abundant in oral and fecal samples of colorectal cancer positives (CPs) for phylum, family, and species versus colorectal cancer negatives (CNs) [# = CRC-exclusive bacteria, * = significant CRC vs. normal differences]

We conducted a comparison of the frequency of the top 10 microbes that were most abundant CPs, analyzing both fecal and oral samples, in terms of phylum, family, and species in comparison to CN samples (see Fig. 1). Notably, some of these microbes were completely absent in CNs, while others exhibited a significant difference in their presence.

In the saliva of CPs, *Chloroflexi*, *Lactobacillaceae*, *Rivulariaceae*, *Calothrix parietina*, *Rothia dentocariosa*, and *Rothia mucilaginosa* ranked among the top 10 microbes, none of which were present in the saliva of CN individuals. Conversely, in the feces of CRC patients, *Coprobacillaceae*, *Enterococcaceae*, *Neisseriaceae*, *Streptococcaceae*, *Bacteroides cellulosilyticus*, *Coprobacillus cateniformis*, *Porphyromonas asaccharolytica*, *Sphingobacterium bambusae*, and *Streptococcus vestibularis* were identified among the 10 most abundant microbes at the family and species levels, with none of them present in CN participants.

Furthermore, our analysis revealed a higher abundance of microbes such as *Fusobactria* in the saliva of CRC patients compared to CN individuals. Additionally, a significant *p*-value indicated a higher amount of *Lachnospiraceae* and *Prevotellaceae* in the stool of CPs compared to controls, suggesting that these microbes are present in both CNs and CPs, but their quantity is elevated in CPs.

In the Table 2, the median and the *p*-value of these 10 more abundant microbes in the saliva and feces of CRC patients compared to CNs regarding the phylum, family and species have been investigated in detail.

**Table 2:** Median (first quartile, third quartile) and a *p*-value of each individual candidate bacteria based on abundancy

	Type	CRC positives	CRC negatives	*p*-value
*Stool*				
Top family	*Bacteroidaceae*	35,138 (20,473, 57,097)	26,441 (19,861.25, 51,036.5)	0.975
	*Coprobacillaceae*	22,037 (22,037, 22,037)	NA	
	*Enterobacteriaceae*	32,034 (11,517.5, 45,663)	26,693 (12,241, 67,296)	0.961
	*Enterococcaceae*	21,837 (21,837, 21,837)	NA	
	*Lachnospiraceae*	20,466.5 (15,082.75, 26,686.25)	9915 (8118.5, 18,518)	**0.006**
	*Neisseriaceae*	21,907 (21,907, 21,907)	NA	
	*Pasteurellaceae*	19,133.5 (11,658, 26,609)	3078 (3078, 3078)	0.221
	*Prevotellaceae*	18,563 (13,833, 42,279)	71,417 (29,676.5, 106,358)	**0.021**
	*Ruminococcaceae*	20,242 (13,254.25, 23,827)	22,173 (9717.5, 26,917.5)	0.946
	*Streptococcaceae*	20,557 (245, 29,531)	NA	
Top species	*Bacteroides cellulosilyticus*	16,964 (16,964, 16,964)	NA	
	*Bacteroides vulgatus*	13,640 (6536, 26,760.5)	6081 (3432.75, 12,740.5)	0.090
	*Coprobacillus cateniformis*	22,036 (22,036, 22,036)	NA	
	*Escherichia albertii*	14,601 (5602.75, 24,528.5)	12,575 (4297.5, 33,527)	0.752
	*Haemophilus parainfluenzae*	12,201.5 (6813, 17,590)	NA	
	*Porphyromonas asaccharolytica*	20,356 (20,356, 20,356)	NA	
	*Prevotella copri*	16,278 (3795, 24,755)	21,376 (3760, 57,922)	0.441
	*Sphingobacterium bambusae*	14,383 (14,383, 14,383)	NA	
	*Streptococcus vestibularis*	12,767 (12,767, 12,767)	NA	
	*Unclassified at Species level*	70,268 (58,857.5, 79,222)	65,762 (57,502.75, 87,313.5)	0.639
Top phylum	Actinobacteria	900 (396, 2108.5)	1271.5 (415.75, 2160.75)	0.770
	Bacteroidetes	82,999 (32,927, 102,132.5)	89,034 (56,220.75, 104,966.25)	0.429
	Firmicutes	58,043 (47,733, 72,777.5)	56,212 (38,746, 74,629.75)	0.725
	Fusobacteria	788.5 (271.5, 2717.75)	13 (13, 13)	0.114
	Nitrospirae	325.5 (142, 1003.25)	133 (63, 616.5)	0.124
	Proteobacteria	16,907 (11,310.5, 44,244.5)	9314 (7409.25, 26,199.5)	0.053
	Spirochaetes	307.5 (118, 497)	NA	
	Tenericutes	226 (41, 1221.5)	1262 (22, 2502)	1.000
	Unclassified at Phylum level	1884 (1154.5, 2477.5)	1767.5 (1278.5, 2876.75)	0.930
	Verrucomicrobia	958.5 (271.25, 2273)	210 (82.75, 9329.75)	0.269
*Saliva*				
Top family	*Flavobacteriaceae*	6784.5 (2604.5, 8936.5)	8128 (7083, 10,112)	0.289
	*Gemellaceae*	9110 (2739, 9895)	3722 (2054, 5392)	0.210
	*Lactobacillaceae*	16,178 (5758, 25,449)	NA	
	*Micrococcaceae*	10,796 (6851, 22,256)	5363 (5363, 5363)	0.384
	*Neisseriaceae*	27,814 (6188, 41,674)	22,459 (9471.5, 30,426)	0.695
	*Porphyromonadaceae*	9893 (5834, 19,547.5)	6259.5 (3340.25, 9906.75)	0.093
	*Prevotellaceae*	30,879 (9420.5, 39,816.5)	20,346 (8663, 31,362)	0.386
	*Rivulariaceae*	52,390 (4881, 99,899)	NA	
	*Streptococcaceae*	37,934 (28,412, 47,562)	52,361 (33,431, 62,602)	0.156
	*Veillonellaceae*	24,049.5 (9543.75, 36,522.5)	26,681 (17,955, 34,776)	0.536
Top species	*Calothrix parietina*	52,381.5 (4878, 99,885)	NA	
	*Granulicatella adiacens*	11,447 (5475, 11,609)	NA	
	*Neisseria flavescens*	11,156 (6278, 16,034)	10,976.5 (7555.75, 14,895.25)	1.000
	*Neisseria mucosa*	15,137 (9509.25, 29,842.25)	10,501 (8133, 16,335)	0.288
	*Neisseria subflava*	12,388 (12,388, 12,388)	4749 (4749, 4749)	0.317
	*Prevotella melaninogenica*	16,095 (12,500.5, 17,582)	12,400 (6664, 17,685)	0.273
	*Rothia dentocariosa*	10,847 (10,847, 10,847)	NA	
	*Rothia mucilaginosa*	13,025.5 (5100.5, 20,090.75)	NA	
	*Unclassified at Species level*	47,894 (43,544, 57,892)	48,100 (44,866, 54,567)	0.846
	*Veillonella atypica*	11,240 (8108, 15,829.75)	11,364 (8764, 17,463)	0.767
Top phylum	Actinobacteria	8467 (2359, 17,695)	4542 (3117, 6773)	0.097
	Bacteroidetes	43,222.5 (23,659.25, 52,389.25)	30,558 (20,851, 43,199)	0.279
	Chloroflexi	1037 (1037, 1037)	NA	
	Firmicutes	76,819 (68,534, 96,665)	100,930 (72,165, 107,327)	0.263
	Fusobacteria	3328 (2133, 5205)	5466 (4048, 7725)	**0.018**
	Proteobacteria	15,284 (7899, 53,296)	31,032 (11,128, 55,320)	0.317
	Spirochaetes	146 (111.25, 1249.5)	190 (112.5, 304.5)	0.847
	Thermi	161.5 (64.75, 232)	NA	
	Unclassified at Phylum level	1131 (930, 1279)	973 (807, 1028)	0.135
	Verrucomicrobia	221 (91, 328.5)	134.5 (87.75, 242)	0.495

The median [interquartile range (IQR)] was reported for each bacterium. The Dunn’s test was used to test the median of each bacterium between CRC and Normal. NA means that some microbiotas do not have third quartile values

Significant *p*-values were bolded

#### Non-pathogenic microbiota

**Fig. 2:**
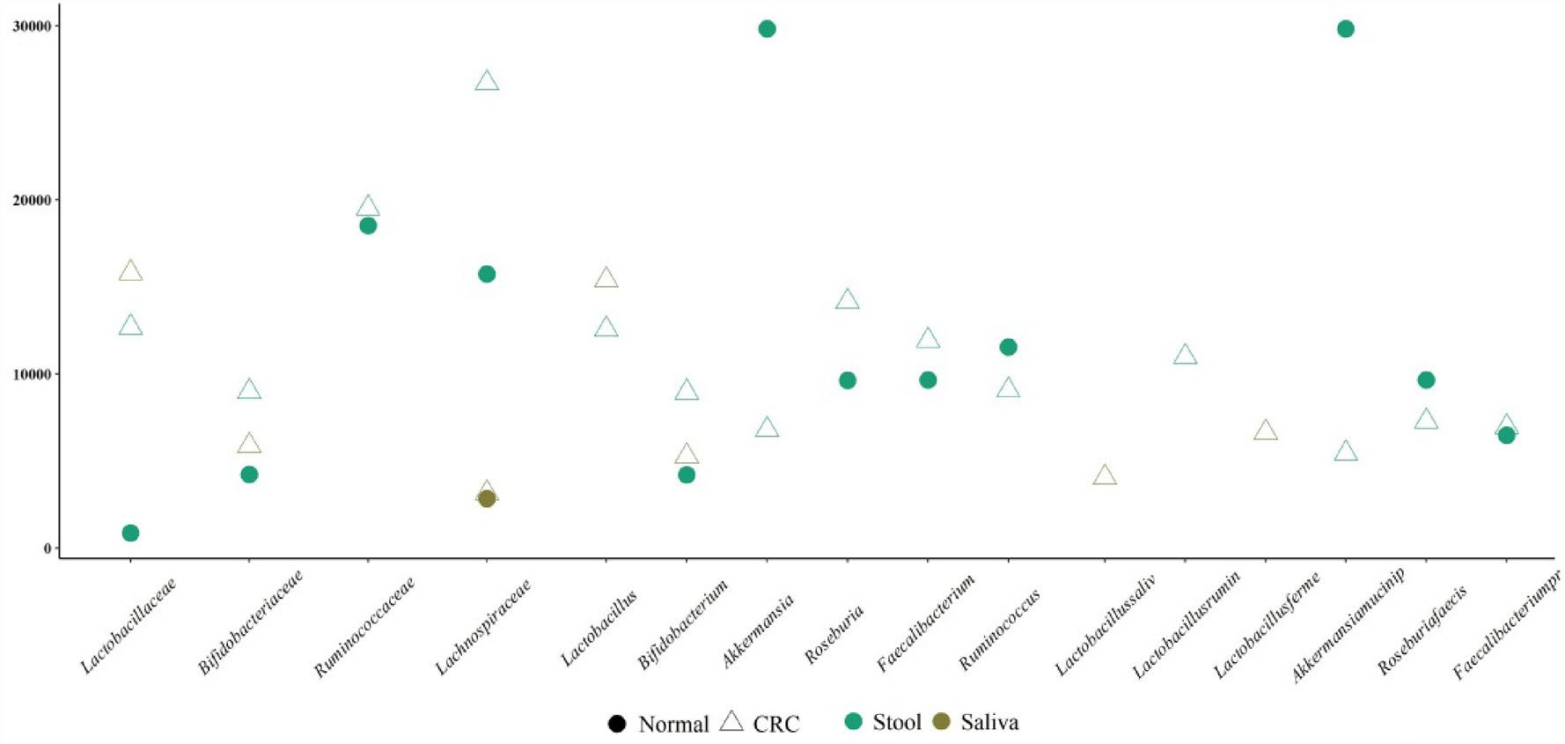
The higher abundancy of the genus *Akkermansia* and the species *Akkermansia muciniphila* among all the non-pathogenic microbes in the stool samples of colorectal cancer negatives versus colorectal cancer positive patients

An investigation into a range of commensal microbiota, including *Lactobacillaceae*, *Bifidobacteriaceae*, *Ruminococcaceae*, *Lachnospiraceae*, *Lactobacillus*, *Bifidobacterium*, *Akkermansia*, *Roseburia*, *Faecalibacterium*, and *Ruminococcus,* was conducted in the feces of CPs in comparison to CNs (see Fig. 2). Notably, among all the non-pathogenic microbes analyzed in the stool samples, the genus *Akkermansia* and the species *Akkermansia muciniphila* were significantly more abundant in the CN group than in CRC patients.

Based on microbial variables that have the least missing data, 24 microbes in saliva and 27 microbes in stool were selected. AUROC, sensitivity, specificity, PPV, NPV and ACC were calculated for each bacterium. For ROC analysis, four different models were used, including logistic regression, support vector machine, naïve bayes and neural network. In Table 3 we showed which microbes are most important in predicting CRC. Four of them in saliva have the highest AUC which include *Porphyromonadaceae*, Unclassified at Family level, *Fusobacteria*, and *Streptococcus infantis*. Also, four of the microbes in stool have the highest AUC, which include *Lachnospiraceae*, *Proteobacteria*, *Nitrospirae* and *Escherichia albertii*. Confidence interval (CI) was reported for SE, SP, PPV, NPV and ACC.

**Table 3:** The Prediction performance using logistic regression for each microbiota

	Variables	AUC (95% CI)	Cut-off	SE (95% CI)	SP (95% CI)	PPV (95% CI)	NPV (95% CI)	Accuracy (95% CI)
Saliva	Neisseriaceae	0.57 (0.38, 0.76)	0.61943	0.61 (0.39, 0.80)	0.67 (0.38, 0.88)	0.74 (0.47, 0.88)	0.53 (0.31, 0.81)	0.61 (0.43, 0.76)
	Prevotellaceae	0.59 (0.41, 0.78)	0.66244	0.48 (0.27, 0.69)	0.80 (0.52, 0.96)	0.79 (0.50, 0.90)	0.50 (0.29, 0.85)	0.58 (0.41, 0.74)
	Streptococcaceae	0.64 (0.44, 0.83)	0.56746	0.87 (0.66, 0.97)	0.53 (0.27, 0.79)	0.74 (0.48, 0.94)	0.73 (0.44, 0.90)	0.71 (0.54, 0.85)
	Veillonellaceae	0.54 (0.35, 0.73)	0.60961	0.26 (0.10, 0.48)	0.93 (0.68, 1.00)	0.86 (0.48, 0.94)	0.45 (0.21, 0.97)	0.50 (0.33, 0.67)
	Unclassified at family level	**0.70 (0.53, 0.87)**	0.60513	0.61 (0.39, 0.80)	0.87 (0.60, 0.98)	0.88 (0.61, 0.95)	0.59 (0.37, 0.93)	0.71 (0.54, 0.85)
	Pasteurellaceae	0.55 (0.35, 0.75)	0.63407	0.65 (0.43, 0.84)	0.53 (0.27, 0.79)	0.68 (0.40, 0.85)	0.50 (0.28, 0.76)	0.53 (0.36, 0.69)
	Porphyromonadaceae	**0.74 (0.58, 0.90)**	0.60168	0.57 (0.34, 0.77)	0.80 (0.52, 0.96)	0.81 (0.54, 0.92)	0.55 (0.33, 0.87)	0.61 (0.43, 0.76)
	Firmicutes	0.61 (0.42, 0.80)	0.57158	0.78 (0.56, 0.93)	0.53 (0.27, 0.79)	0.72 (0.45, 0.90)	0.62 (0.36, 0.84)	0.68 (0.51, 0.82)
	Proteobacteria	0.60 (0.41, 0.78)	0.62610	0.65 (0.43, 0.84)	0.60 (0.32, 0.84)	0.71 (0.44, 0.87)	0.53 (0.31, 0.79)	0.63 (0.46, 0.78)
	Bacteroidetes	0.60 (0.41, 0.78)	0.62585	0.57 (0.34, 0.77)	0.73 (0.45, 0.92)	0.76 (0.49, 0.89)	0.52 (0.31, 0.83)	0.63 (0.46, 0.78)
	Actinobacteria	0.66 (0.48, 0.84)	0.65617	0.52 (0.31, 0.73)	0.93 (0.68, 1.00)	0.92 (0.65, 0.97)	0.56 (0.34, 0.98)	0.68 (0.51, 0.82)
	Fusobacteria	**0.73 (0.57, 0.89)**	0.64114	0.65 (0.43, 0.84)	0.80 (0.52, 0.96)	0.83 (0.57, 0.93)	0.60 (0.37, 0.89)	0.68 (0.51, 0.82)
	Unclassified at phylum level	0.64 (0.47, 0.82)	0.62979	0.57 (0.34, 0.77)	0.80 (0.52, 0.96)	0.81 (0.54, 0.92)	0.55 (0.33, 0.87)	0.63 (0.46, 0.78)
	Verrucomicrobia	0.44 (0.24, 0.64)	0.58570	0.91 (0.72, 0.99)	0.20 (0.04, 0.48)	0.64 (0.24, 0.94)	0.60 (0.27, 0.85)	0.61 (0.43, 0.76)
	Cyanobacteria	0.45 (0.26, 0.64)	0.61070	0.13 (0.03, 0.34)	1.00 (0.78, 1.00)	1.00 (0.42, 1.00)	0.43 (0.12, 1.00)	0.47 (0.31, 0.64)
	Spirochaetes	0.45 (0.25, 0.65)	0.57570	1.00 (0.85, 1.00)	0.20 (0.04, 0.48)	0.66 (0.26, 1.00)	1.00 (0.43, 1.00)	0.58 (0.41, 0.74)
	Unclassified at species level	0.52 (0.33, 0.71)	0.61704	0.43 (0.23, 0.66)	0.80 (0.52, 0.96)	0.77 (0.47, 0.89)	0.48 (0.27, 0.84)	0.55 (0.38, 0.71)
	Neisseria mucosa	0.61 (0.43, 0.79)	0.62068	0.57 (0.34, 0.77)	0.80 (0.52, 0.96)	0.81 (0.54, 0.92)	0.55 (0.33, 0.87)	0.58 (0.41, 0.74)
	Prevotella melaninogenica	0.66 (0.48, 0.85)	0.58381	0.70 (0.47, 0.87)	0.60 (0.32, 0.84)	0.73 (0.46, 0.88)	0.56 (0.33, 0.81)	0.66 (0.49, 0.80)
	Veillonella atypica	0.49 (0.29, 0.69)	0.59292	0.91 (0.72, 0.99)	0.27 (0.08, 0.55)	0.66 (0.31, 0.94)	0.67 (0.33, 0.87)	0.63 (0.46, 0.78)
	Veillonella dispar	0.56 (0.37, 0.75)	0.59037	0.52 (0.31, 0.73)	0.67 (0.38, 0.88)	0.71 (0.43, 0.86)	0.48 (0.27, 0.77)	0.53 (0.36, 0.69)
	Streptococcus tigurinus	0.67 (0.49, 0.86)	0.69117	0.52 (0.31, 0.73)	0.87 (0.60, 0.98)	0.86 (0.58, 0.94)	0.54 (0.32, 0.92)	0.63 (0.46, 0.78)
	Streptococcus pseudopneumoniae	0.67 (0.49, 0.84)	0.72796	0.30 (0.13, 0.53)	1.00 (0.78, 1.00)	1.00 (0.63, 1.00)	0.48 (0.25, 1.00)	0.53 (0.36, 0.69)
	Streptococcus infantis	**0.74 (0.57, 0.90)**	0.73146	0.61 (0.39, 0.80)	0.87 (0.60, 0.98)	0.88 (0.61, 0.95)	0.59 (0.37, 0.93)	0.71 (0.54, 0.85)
Stool	Prevotellaceae	0.45 (0.24, 0.66)	0.59528	1.00 (0.86, 1.00)	0.21 (0.05, 0.51)	0.69 (0.29, 1.00)	1.00 (0.43, 1.00)	0.72 (0.55, 0.85)
	Lachnospiraceae	**0.70 (0.50, 0.89)**	0.60186	0.88 (0.69, 0.97)	0.57 (0.29, 0.82)	0.79 (0.53, 0.95)	0.73 (0.44, 0.90)	0.77 (0.61, 0.89)
	Ruminococcaceae	0.48 (0.26, 0.69)	0.63412	0.80 (0.59, 0.93)	0.43 (0.18, 0.71)	0.71 (0.42, 0.90)	0.55 (0.30, 0.80)	0.67 (0.50, 0.81)
	Bacteroidaceae	0.54 (0.34, 0.73)	0.67614	0.28 (0.12, 0.49)	0.93 (0.66, 1.00)	0.88 (0.51, 0.95)	0.42 (0.20, 0.97)	0.51 (0.35, 0.68)
	Unclassified at family level	0.53 (0.34, 0.72)	0.64502	0.52 (0.31, 0.72)	0.64 (0.35, 0.87)	0.72 (0.44, 0.86)	0.43 (0.24, 0.74)	0.56 (0.40, 0.72)
	Enterobacteriaceae	0.60 (0.42, 0.79)	0.68041	0.40 (0.21, 0.61)	0.86 (0.57, 0.98)	0.83 (0.53, 0.92)	0.44 (0.24, 0.88)	0.56 (0.40, 0.72)
	Clostridiaceae	0.57 (0.37, 0.77)	0.63297	0.72 (0.51, 0.88)	0.50 (0.23, 0.77)	0.72 (0.43, 0.88)	0.50 (0.28, 0.77)	0.64 (0.47, 0.79)
	Flavobacteriaceae	0.54 (0.34, 0.73)	0.65106	0.44 (0.24, 0.65)	0.79 (0.49, 0.95)	0.79 (0.49, 0.90)	0.44 (0.24, 0.81)	0.44 (0.28, 0.60)
	Alcaligenaceae	0.57 (0.39, 0.76)	0.95574	0.36 (0.18, 0.57)	1.00 (0.77, 1.00)	1.00 (0.68, 1.00)	0.47 (0.25, 1.00)	0.49 (0.32, 0.65)
	Porphyromonadaceae	0.52 (0.33, 0.70)	0.68017	0.32 (0.15, 0.54)	0.93 (0.66, 1.00)	0.89 (0.55, 0.95)	0.43 (0.22, 0.97)	0.54 (0.37, 0.70)
	Veillonellaceae	0.60 (0.40, 0.81)	0.63125	0.84 (0.64, 0.95)	0.43 (0.18, 0.71)	0.72 (0.43, 0.91)	0.60 (0.34, 0.83)	0.69 (0.52, 0.83)
	Bacteroidetes	0.58 (0.39, 0.77)	0.68012	0.40 (0.21, 0.61)	0.79 (0.49, 0.95)	0.77 (0.47, 0.89)	0.42 (0.23, 0.80)	0.51 (0.35, 0.68)
	Firmicutes	0.53 (0.33, 0.74)	0.63277	0.92 (0.74, 0.99)	0.29 (0.08, 0.58)	0.70 (0.34, 0.95)	0.67 (0.33, 0.87)	0.67 (0.50, 0.81)
	Proteobacteria	0.69 (0.50, 0.88)	0.57792	0.80 (0.59, 0.93)	0.64 (0.35, 0.87)	0.80 (0.55, 0.93)	0.64 (0.40, 0.87)	0.72 (0.55, 0.85)
	Unclassified at phylum level	0.51 (0.31, 0.71)	0.63133	0.80 (0.59, 0.93)	0.36 (0.13, 0.65)	0.69 (0.37, 0.88)	0.50 (0.27, 0.77)	0.64 (0.47, 0.79)
	Actinobacteria	0.47 (0.28, 0.66)	0.70535	0.12 (0.03, 0.31)	1.00 (0.77, 1.00)	1.00 (0.42, 1.00)	0.39 (0.11, 1.00)	0.41 (0.26, 0.58)
	Nitrospirae	0.64 (0.44, 0.84)	0.56291	1.00 (0.86, 1.00)	0.29 (0.08, 0.58)	0.71 (0.36, 1.00)	1.00 (0.50, 1.00)	0.74 (0.58, 0.87)
	Cyanobacteria	0.44 (0.24, 0.63)	0.68098	0.12 (0.03, 0.31)	1.00 (0.77, 1.00)	1.00 (0.42, 1.00)	0.39 (0.11, 1.00)	0.44 (0.28, 0.60)
	Chloroflexi	0.60 (0.42, 0.79)	0.59631	0.64 (0.43, 0.82)	0.64 (0.35, 0.87)	0.76 (0.49, 0.89)	0.50 (0.29, 0.79)	0.62 (0.45, 0.77)
	Verrucomicrobia	0.48 (0.28, 0.68)	0.64675	0.68 (0.46, 0.85)	0.43 (0.18, 0.71)	0.68 (0.38, 0.85)	0.43 (0.23, 0.71)	0.59 (0.42, 0.74)
	Unclassified at species level	0.55 (0.34, 0.75)	0.64183	0.60 (0.39, 0.79)	0.64 (0.35, 0.87)	0.75 (0.47, 0.88)	0.47 (0.27, 0.77)	0.59 (0.42, 0.74)
	Prevotella copri	0.46 (0.26, 0.67)	0.61832	1.00 (0.86, 1.00)	0.07 (0.00, 0.34)	0.66 (0.04, 1.00)	1.00 (0.20, 1.00)	0.64 (0.47, 0.79)
	Bacteroides vulgatus	0.55 (0.35, 0.76)	0.64071	0.56 (0.35, 0.76)	0.64 (0.35, 0.87)	0.74 (0.46, 0.87)	0.45 (0.26, 0.76)	0.56 (0.40, 0.72)
	Escherichia albertii	0.64 (0.45, 0.83)	0.55030	0.80 (0.59, 0.93)	0.50 (0.23, 0.77)	0.74 (0.46, 0.91)	0.58 (0.34, 0.82)	0.67 (0.50, 0.81)
	Bacteroides fragilis	0.56 (0.36, 0.76)	0.66099	0.48 (0.28, 0.69)	0.79 (0.49, 0.95)	0.80 (0.51, 0.90)	0.46 (0.26, 0.83)	0.51 (0.35, 0.68)

Higher than 0.7 for AUCs were selected and bolded

**Fig. 3:**
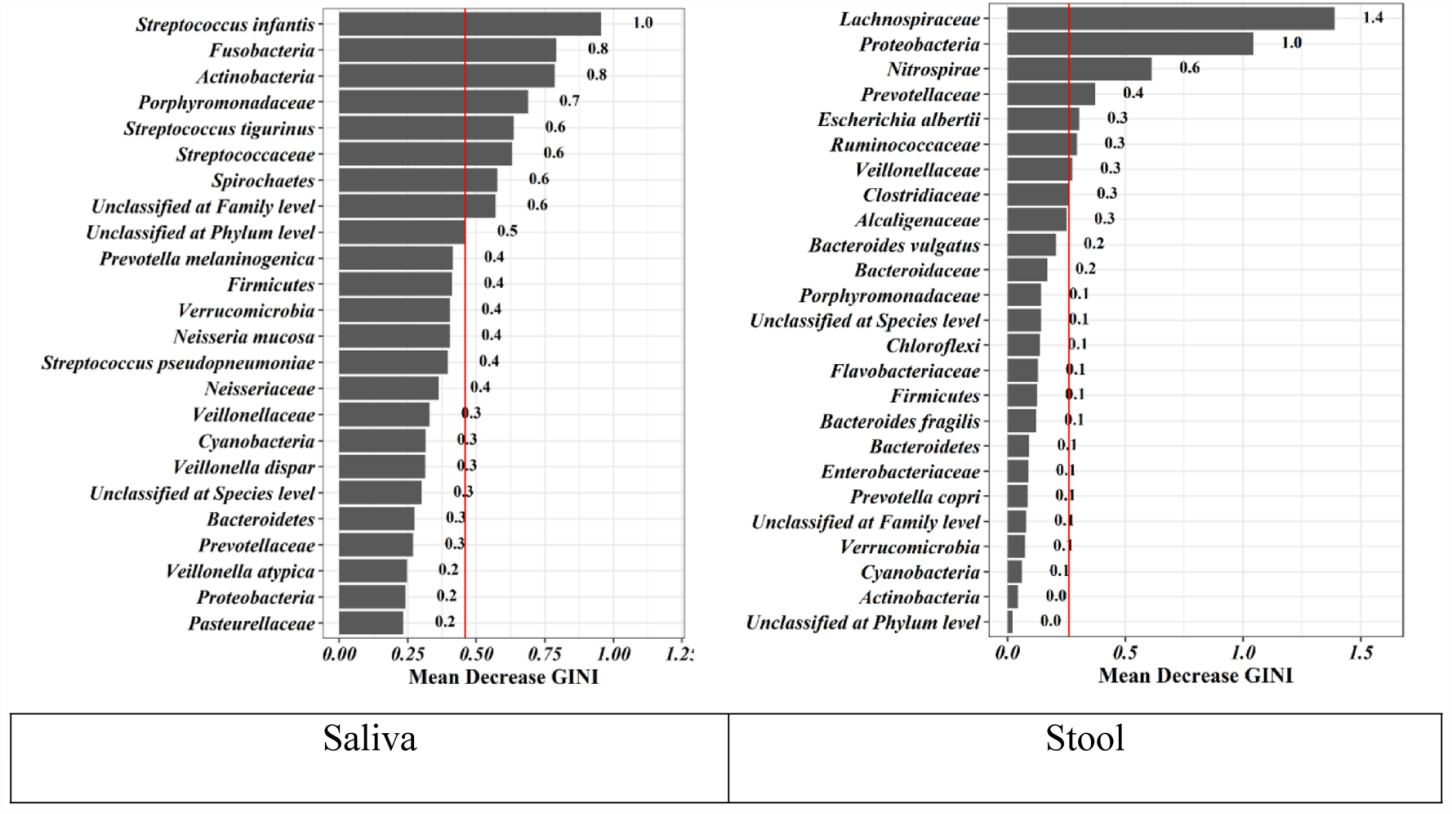
Mean Decrease GINI model for colorectal cancer prediction. Higher mean decreases in GINI for bacteria show that bacteria are more important in predicting CRC. *The Mean Decrease GINI presents those microbes that have the highest amount in GINI, their removal makes the model worse in the direction of predicting CRC and their presence helps the model to be powerful

In Fig. 3, important microbes in predicting CRC in saliva include *Streptococcus infantis*, *Fusobacteria*, *Actinobacteria*, *Porphyromonadaceae*, *Streptococcus tigurinus*, *Streptococcaceae, Spirochaetes*, Unclassified at Family level, and Unclassified at phylum level. Also, important microbes in predicting CRC in stool include *Lachnospiraceae*, *Proteobacteria*, *Nitrospirae*, *Prevotellaceae*, *Escherichia albertii*, Ruminococcaceae, *Veillonellaceae*, *Clostridiaceae*, and *Alcaligenaceae.*

### Combination of selected variable microbiota based on mean decrease GINI model for improvement of the diagnostic ability for early detection of CRC

The desired microbial variables were selected based on Mean Decrease GINI, and then we examined multiple regressions. Multiple regressions mean to use certain microbiota simultaneously in certain statistical models to predict CRC patients. Four different models including logistic regression, support vector machine, Naïve Bayes, neural network were selected along with a selection of microbiota based on GINI. For saliva, the logistic model is the best model among others due to its simplicity and AUC of 91%, SE of 87%, SP of 80%, PPV 87%, NPV of 80% and ACC of 84% (Table 4). For stool, the support vector machine was the best model because it has performed with the highest AUC of 97%, SE of 92%, SP of 93%, PPV of 96%, NPV of 87% and ACC of 90% compared to other models, even the simple logistic regression (Table 4).

**Table 4:** The Prediction performance using logistic regression with selected variables for each microbiota

	Variables	AUC (95% CI)	Cut-off	SE (95% CI)	SP (95% CI)	PPV (95% CI)	NPV (95% CI)	Accuracy (95% CI)
Saliva	Logistic-total variables	1.00 (1.00, 1.00)	1.00	1.00 (0.85, 1.00)	1.00 (0.78, 1.00)	1.00 (0.85, 1.00)	1.00 (0.79, 1.00)	0.39 (0.24, 0.57)
	Logistic-selected variable	0.91 (0.82, 1.00)	0.60	0.87 (0.66, 0.97)	0.80 (0.52, 0.96)	0.87 (0.64, 0.97)	0.80 (0.54, 0.96)	0.84 (0.69, 0.94)
	Support vector machine	0.90 (0.77, 1.00)	0.57	0.91 (0.72, 0.99)	0.87 (0.60, 0.98)	0.91 (0.70, 0.99)	0.87 (0.61, 0.98)	0.87 (0.72, 0.96)
	Naïve Bayes	0.93 (0.84, 1.00)	0.10	0.91 (0.72, 0.99)	0.87 (0.60, 0.98)	0.91 (0.70, 0.99)	0.87 (0.61, 0.98)	0.89 (0.75, 0.97)
	Neural network	0.64 (0.44, 0.83)	0.56	0.87 (0.66, 0.97)	0.53 (0.27, 0.79)	0.74 (0.48, 0.94)	0.73 (0.44, 0.90)	0.71 (0.54, 0.85)
Stool	Logistic-total variables	1.00 (1.00, 1.00)	1.00	1.00 (0.86, 1.00)	1.00 (0.77, NA)	1.00 (0.86, 1.00)	1.00 (0.78, 1.00)	0.36 (0.21, 0.53)
	Logistic-selected variables	0.77 (0.59, 0.96)	0.58	0.76 (0.55, 0.91)	0.79 (0.49, 0.95)	0.86 (0.63, 0.95)	0.65 (0.41, 0.91)	0.77 (0.61, 0.89)
	Support vector machine	0.97 (0.92, 1.00)	0.67	0.92 (0.74, 0.99)	0.93 (0.66, 1.00)	0.96 (0.78, 1.00)	0.87 (0.62, 1.00)	0.90 (0.76, 0.97)
	Naïve Bayes	0.78 (0.61, 0.94)	0.76	0.60 (0.39, 0.79)	0.93 (0.66, 1.00)	0.94 (0.69, 0.97)	0.57 (0.35, 0.98)	0.69 (0.52, 0.83)
	Neural network	0.45 (0.24, 0.66)	0.61	1.00 (0.86, 1.00)	0.21 (0.05, 0.51)	0.69 (0.29, 1.00)	1.00 (0.43, 1.00)	0.69 (0.52, 0.83)

**Fig. 4:**
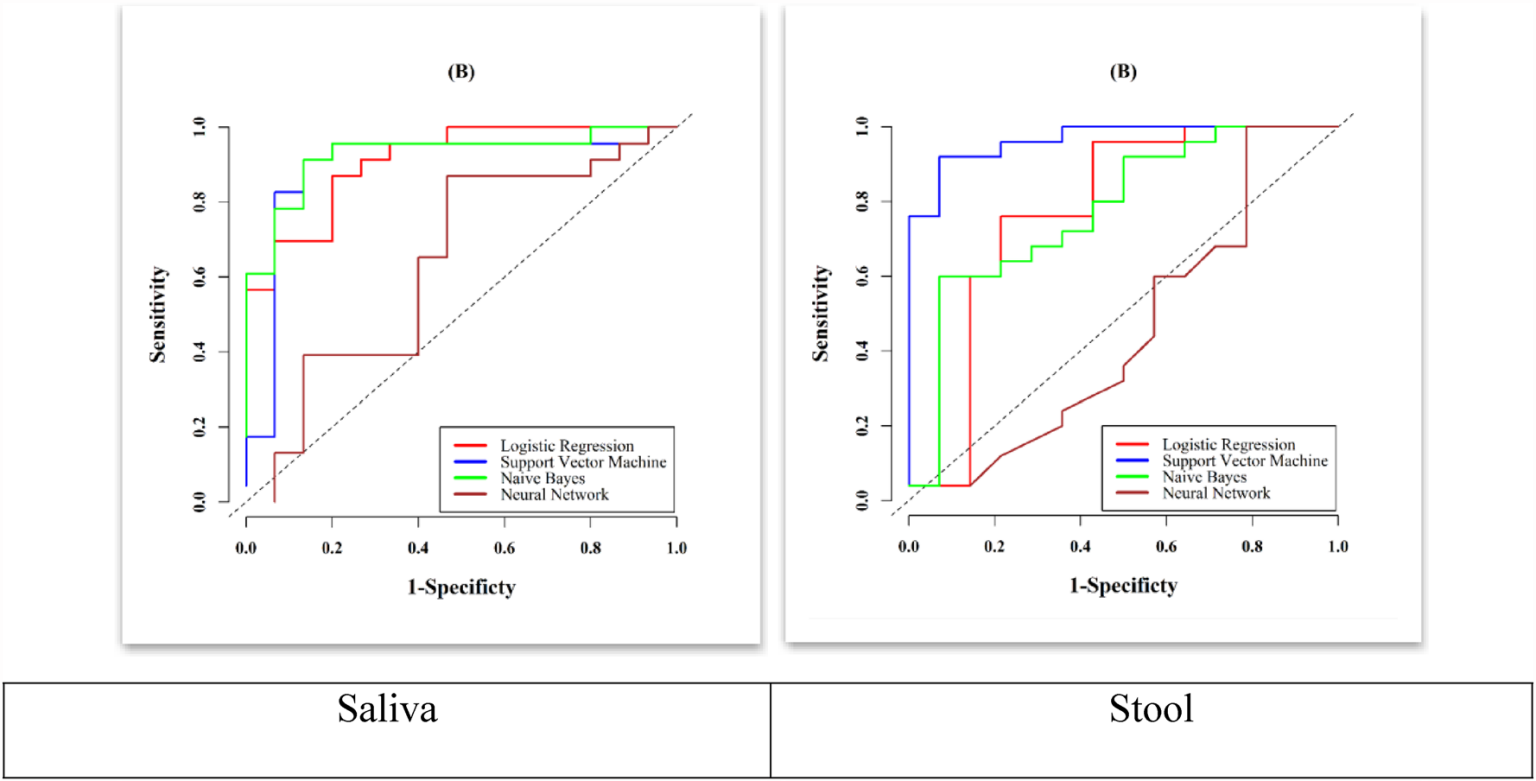
ROC curves with performance of logistic model, support vector machine, naïve bayes and neural network models using selected variables

ROC curves with performance of logistic regression, support vector machine, naïve Bayes and neural network models along with a selection of microbiota based on mean decrease GINI were demonstrated in Fig. 4. At the best cutoff value, this panel of bacteria could be used to discriminate CP patients from CN individuals.

## Discussion

In this study, we conducted the first-ever examination of the integrated microbiome from stool and saliva samples of colorectal cancer (CRC) patients in comparison to healthy controls (CNs) within the Iranian population, utilizing the 16S rRNA sequencing method. The utilization of microbiota as biomarkers for disease and health has gained significant traction, particularly with the advancements in 16S rRNA sequencing technology.

Our results, as depicted in the demographic table, reveal a noteworthy difference between CPs and CNs concerning occupation, physical activity, and smoking habits. Interestingly, housewives and retired individuals exhibited a higher prevalence of CRC compared to working and non-retired individuals. Furthermore, smoking and a lack of exercise were more prevalent among CP patients compared to CNs.

In general, the incidence of CRC tends to be higher in individuals over 50 years old, whereas those under 50 years old, who typically undergo screening, are generally healthier. This age-related discrepancy is a noteworthy factor contributing to the differences observed between the CP and CN groups. Additionally, the occurrence of CRC in individuals with a family history of the disease and a personal history of other illnesses and surgeries was more prevalent than in CNs. This implies that individuals with a susceptibility marked by a history of other diseases and surgeries are more predisposed to CRC than those without such histories.

The notable observation of distinct microbial profiles between CPs and CNs highlights a significant aspect, suggesting that the microbiome may play a crucial role in the initiation and development of CRC. For instance, certain microbial patterns were found to be significantly more abundant in CRC patients compared to CNs, with specific examples including *Chloroflexi*, *Lactobacillaceae*, *Rivulariaceae*, *Calothrix parietina*, *Rothia dentocariosa*, and *Rothia mucilaginosa*, which exhibited higher abundancy in the saliva of CRC patients but were entirely absent in CN individuals. Similarly, *Coprobacillaceae*, *Enterococcaceae*, *Neisseriaceae*, *Streptococcaceae*, *Bacteroides cellulosilyticus*, *Coprobacillus cateniformis*, *Porphyromonas asaccharolytica*, *Sphingobacterium bambusae*, and *Streptococcus vestibularis* were identified as the most abundant microbes in the feces of CRC patients, whereas they were absent in CN individuals.

While our findings suggest a compelling association between the presence or absence of certain microbes and CRC, it is essential to conduct studies on a larger population to provide more definitive insights. Our results align with the research by Flemer et al. [18], who identified 63 operational taxonomic units (OTU) distinguishing CRC cases from CNs, including 29 oral OTU and 34 stool OTU. Additionally, our findings are consistent with previous studies that have highlighted the ability of specific microbiota to differentiate individuals with CRC or adenoma polyps from healthy individuals.

Notably, research conducted across various geographical regions such as the USA, Canada, Ireland, Spain, China, Colorado, France, and India has explored the increased presence of bacteria in CRC. Despite differences in ethnicity and geography influencing microbial patterns, it is intriguing that many of the microbes identified in these studies closely correlate with those increased in our CRC patients, including *Fusobacterium*, *Porphyromonas*, *Prevotella*, *Bacteroides*, and *Streptococcus* [18, 22–28].

Identifying a group of microbes with higher abundance in CPs than in healthy CNs and demonstrating statistical significance is crucial, as it facilitates the selection of potential biomarker candidates. In our study, we observed an increased number of *Fusobacteria* in the saliva of CRC patients compared to CNs, as well as a higher abundance of *Lachnospiraceae* and *Prevotellaceae* in the stool of CRC patients compared to CNs. Consistent with our findings, Flemer et al. reported differential abundance of certain oral microbiotas between CPs and CNs, including *Parvimonas*, *Haemophilus*, *Prevotella, Alloprevotella*, *Neisseria*, *Lachnoanaerobaculum*, and *Streptococcus* [18].

Furthermore, non-pathogenic microbiota in the human gut or microbiota that produces short-chain fatty acids (SCFA) play a crucial role in human health and disease prevention [29]. In our research, *Akkermansia muciniphila* showed significantly higher abundance in CNs compared to CPs. *Akkermansia muciniphila* is an important bacterium that degrades mucin in the gut, and its role is debated regarding whether it is beneficial or harmful [30]. Patients with conditions such as overweight, obesity, type 2 diabetes [31], and inflammatory bowel disease (ulcerative colitis and Crohn's disease) [33, 34] have exhibited reduced levels of *Akkermansia muciniphila* in their intestines. In contrast to our findings, Wang et al. reported that *Akkermansia muciniphila* exacerbated the development of colitis-associated CRC in mice [35]. However, similar to our study, Gu et al. concluded that an increased number of *Akkermansia muciniphila* is associated with protection against inflammatory bowel disease (IBD) and CRC following interventions with nutrients, prebiotics, probiotics, and medications [36]. They noted that despite these therapeutic benefits, some animal studies, such as Wang et al.'s experiment, have reported a negative association with *Akkermansia muciniphila* [35, 36]. Therefore, it is advisable to consider *Akkermansia muciniphila* as both a "friend and foe" until additional research and clinical examinations provide further clarity.

A limitation of this study is the small sample size of the cohort, which lacks geographical coverage and broader applicability of the microbiome-based biomarker approach. Validation and confirmation of these findings would benefit from a larger population. Additionally, there is an age difference between the CPs and CNs, which we have attempted to minimize for future studies.

Furthermore, utilizing a combination of selected variable microbiota based on the Mean Decrease GINI model platform, we aimed to enhance the diagnostic ability for the early detection of CRC. For saliva, logistic regression emerged as the optimal model due to its simplicity, boasting an AUC of 91%, sensitivity of 87%, specificity of 80%, PPV of 87%, NPV of 80%, and an ACC of 84%. In contrast, for stool, the support vector machine outperformed other models, achieving the highest AUC of 97%, sensitivity of 92%, specificity of 93%, PPV of 96%, NPV of 87%, and ACC of 90%.

In previous studies, we examined fecal samples of CRC and polyps’ cases versus normal individuals in the Iranian population, employing three models of logistic regression, simple linear combination, and factor with the q-PCR method, ultimately determining specific biomarkers [15]. We identified elevated counts of *F. nucleatum*, *Enterococcus faecalis*, *Streptococcus bovis*, Enterotoxigenic *Bacteroides fragilis*, and *Porphyromonas* spp. in CRC stages 0 and I, as well as in adenoma polyps’ cases, specifically in tubular adenomas and notably in villous and tubovillous adenomas. This contrasts with samples from normal, hyperplastic, and sessile serrated adenoma groups.

However, in the current study, we investigated the entire fecal and saliva microbiota of CRC patients and CNs in the Iranian population using the 16S rRNA sequencing technique. Statistical modeling was not limited to stool but extended to saliva as well. Sensitivity and specificity were determined, and biomarker candidates were selected. In parallel with our study, Flemer et al. [18] identified 16 oral microbiota OTUs that distinguished CRC patients from CN individuals with a sensitivity of 53% and specificity of 96%. Their model's sensitivity to using fecal microbiota to distinguish CRC patients was 22% with a specificity of 95%. However, with the combination of oral and stool microbiota, the model's sensitivity increased to 76% for CRC detection.

Furthermore, an identical set of biomarkers between our study and the studies of Yuan et al., Deng et al., and Choi et al. included *Bacteroides*, *Prevotella*, *Fusobacterium nucleatum*, and *Veillonella dispar* [37–39]. By comparing the differences and similarities between our study and these findings, we emphasize the necessity of investigating a large cohort consisting of different geographical populations of CP and CN individuals from Europe, Asia, and America to comprehensively compare the microbiome.

## Conclusion

Our findings indicate that both oral and fecal microbiota have the potential to differentiate individuals with CPs from CNs. Additionally, our study revealed a reduction in the abundance of *Akkermansia muciniphila* in the stool of patients with CRC. This raises the question of whether these microbes play a crucial role in maintaining health, and their diminished presence may be associated with the pathogenesis of CRC.

Given these observations, further research into the cellular and molecular mechanisms of *Akkermansia muciniphila* is warranted and should be conducted extensively. Moreover, we recommend larger prospective studies that encompass diverse geographical populations with varying diets. These studies should incorporate the analysis of FIT, fecal microbiota, and oral microbiota composition to validate the promising results obtained in our study.

## Methods

### Study population

The current study follows a case–control design, and clinical samples, including saliva and stool (n = 80), were gathered from participants who underwent colonoscopy at Taleghani Hospital in Tehran, Iran, between 2020 and 2021. All participants volunteered to take part in the study, and samples were obtained prior to the colonoscopy procedure. Those enrolled in the study presented symptoms such as rectal bleeding, changes in bowel movements, abdominal pains, and anemia, prompting their initial screening. CN individuals also underwent the screening test, and their colonoscopy results indicated normal findings. The inclusion and exclusion criteria are thoroughly detailed in our recently published article [16]. Additionally, demographic information for the studied groups was collected through questionnaire forms.

### Stool and saliva samples collection, storage, and extraction

Fecal samples were collected before colonoscopy, at a point when the gut microbiota had returned to baseline levels [15, 20]. These stool samples were preserved at − 80 °C at Taleghani Hospitals until subsequent analysis. Similarly, saliva samples were stored at − 80 °C until utilized in the experiments. The comprehensive protocol for sample collection has been detailed in our prior study [16].

Patients underwent diagnosis through colonoscopy and histopathological review of any biopsy. For oral specimens, thawing was done on ice, and Genomic DNA was extracted using the QIAamp DNA Microbiome Kit from Qiagen (Hilden, Germany). In parallel, stool specimens were thawed, and DNA extraction was carried out using the QIAamp DNA Fecal Mini Kit (Qiagen), following the procedures explained earlier [21, 22].

### PCR amplification and sequencing

The gene specific sequences applied here target the 16S rRNA V3 and V4 regions using primers: a forward (5′TCGTCGGCAGCGTCAGATGTGTATA AGAGACAGCCTACGGGNGGCWGCAG3′) and a reverse (5′GTCTCGTGGGCTCGGAG ATGTGTATAAGAGACAGGACTACHVGGGTATCTAATCC3′). The 25 µL PCR was set up as follow: 12.5 µL per sample 2xKAPA HiFi HotStart Ready Mix, 5 µL forward primer (1 µM), 5 µL reverse primer (1 µM), and 2.5 µL genomic DNA of bacteria (5 ng/µL in 10 mM Tris pH 8.5). The thermal cycling situation for amplification of PCR was as follows: initial incubation step at 98 °C for 3 min, 30 denaturation cycles at 94 °C for 30 s, annealing step at 55 °C for 30 s, extension at 72 °C for 30 s, and a final extension at 72 °C for 5 min [16]. Then, 1 µL of PCR product was run on a BioanalyzerDNA 1000 chip to verify the size. Using the V3 and V4 primer pairs in current study, the expected size on a Bioanalyzer trace after the Amplicon PCR step is ~ 550 bp. Amplicon product purification was done with AMPure XP beads based on the manufacturer’s protocol to remove contaminants and PCR artifacts. Purified amplicons were utilized to construct the library based on standard protocols, and sequencing was done using the Nextera XT Index Kiton on an Illumina NovaSeq platform (Illumina, San Diego, CA, USA) [16].

Demultiplexed raw sequences were imported into QIIME2 v.2022-2 [40] and were denoised and clustered using DADA2 [41]. Taxonomy classification was done using the pre-trained, via scikit-learn [42], SILVA [43] with 138 99% full-length sequences. The resulting amplicon sequence variant (ASV) table, taxonomy assignment, and appropriate metadata were applied as input for the Marker Data Profiling module of the online platform Microbiome Analyst [44]. Features with low counts (< 4 and < 20% prevalence in samples, n = 1815) along with those with low variance (based on interquartile range, n = 25) were excluded from the downstream analyses counts were normalized using Total Sum Scaling (TSS).

### Statistical analysis

Descriptive statistics were presented using mean ± standard deviation (SD) and median (interquartile range [IQR]) for quantitative data by group (CNs and CPs). The independent t-test was applied to compare the mean of age between CRC and normal groups. The Fisher exact test or exact Pearson Chi-Square was used to evaluate the relation between categorical variables and group. Barplots were utilized to show the frequency of microbiota and compare them between the CPs and CNs groups. The "*" symbol in barplots represents statistically significant differences between CRC samples and normal samples, while the "#" symbol highlights CRC-exclusive bacteria. Analyses were conducted applying SPSS (version 26) and R (version 4.2.1). *p*-values less than 0.05 were assumed as statistically significant.

### Machine learning algorithm

In current study, subjects were randomly divided into two groups: training specimens (70% of samples) and validation specimens (30% of samples). Models were created based on training data and tested based on validation data. It is possible for a patient to appear in only one sample, depending on which sample was used. Data in training was used to expand models including logistic regression (LR), naive baye (NB), support vector machine (SVM), and neural network (NN) [45–47].

### Tune parameters

Each of the methods described here has a number of parameters associated with it, and it is crucial that the most appropriate parameter be selected in order to produce both the optimal and minimal model. In order to accurately predict diseases, each algorithm was fine-tuned. The fivefold cross validation was used with ten iterations to tune each machine learning algorithm, utilizing available statistical codes and R packages.

### Performance evaluation

An area under Receiver Operating Characteristics (ROC) curve (AUC) was used to estimate and compare models, followed by sensitivity, specificity, positive predictive values (PPV), negative predictive values (NPV), and accuracy (ACC). AUC was used as the criteria for selecting the most effective model for clinical decision-making. The ROC curve depicts the sensitivity and specificity of different diagnostic tests. There is no discrimination for example the ability to diagnose cases with or without a disease at AUC 0.5, 0.7–0.8 is acceptable, 0.8–0.9 is excellent, and more than 0.9 is exceptional [48]. Sensitivity is defined as the percentage of patients with the disease predicted in the model to be patients with the disease. The model must be able to nicely recognize all CRC cases in regard to attain 100% sensitivity. The specificity of the model refers to the percentage of cases without the CRC who will be predicted to be CNs as a result of the model. The model should nicely recognize all CNs in order to be 100% specific. PPP refers to the percentage of CRC cases who were speculated to have CRC who really have it. NPV refers to the proportion of individuals speculated as CNs that really do not have CRC. A prediction's ACC is assessed by dividing the number of correct predictions by the number of observations.

### Selection variable

A Random Forest technique was used in regard to characterize the importance of the variable based on the mean decrease in GINI. Higher mean decreases in GINI for gut bacteria show that bacteria are more important in predicting CRC [49]. A fivefold cross-validation method with 10 iterations was applied to tune the parameters of the random forest.

## Data Availability

Most data generated and analyzed during this study is included in this published article. Additional dataset used and/or analyzed during the current study are available from the corresponding author on reasonable request.

## Abbreviations

CRC: Colorectal cancer
CPs: Colorectal cancer positives
CNs: Colorectal cancer negatives
FIT: Fecal immunochemical test
qPCR: Quantitative PCR
CI: Confidence interval
OUT: Operational taxonomic units
SD: Standard deviation
IQR: Interquartile range
LR: Logistic regression models
NB: Naïve Bayes models
SVM: Support vector machines
NN: Neural network models
ROC: Receiver operating characteristics
AUC: Area under curve
PPV: Positive predictive value
NPV: Negative predictive value
ACC: Accuracy
