# Correction to: 16 S rRNA sequencing analysis of the oral and fecal microbiota in colorectal cancer positives versus colorectal cancer negatives in Iranian population

**DOI:** 10.1186/s13099-024-00607-x

**Published:** 2024-03-19

**Authors:** Sama Rezasoltani, Mehdi Azizmohammad Looha, Hamid Asadzadeh Aghdaei, Seyedesomayeh Jasemi, Leonardo Antonio Sechi, Maria Gazouli, Amir Sadeghi, Shirin Torkashvand, Reyhaneh Baniali, Hartmut Schlüter, Mohammad Reza Zali, Mohammad Mehdi Feizabadi

**Affiliations:** 1 Section Mass Spectrometric Proteomics, Diagnostic Center, University Medical Center Hamburg-Eppendorf (UKE), 20246 Hamburg, Germany; 2 Division of Oral Microbiology and Immunology, Department of Operative Dentistry, Periodontology and Preventive Dentistry, RWTH University Hospital, Pauwelsstrasse 30, 52057 Aachen, Germany; 3 Basic and Molecular Epidemiology of Gastrointestinal Disorders Research Center, Research Institute for Gastroenterology and Liver Diseases, Shahid Beheshti University of Medical Sciences, Tehran, 19835-178 Iran; 4 Department of Biomedical Sciences, University of Sassari, Viale San Pietro 43b, Sassari, 07100 Italy; 5 Struttura Complessa Microbiologia e Virologia, Azienda Ospedaliera Universitaria, Sassari, 07100 Italy; 6 Department of Basic Medical Sciences, Laboratory of Biology, Medical School, National and Kapodistrian University of Athens, Athens, Greece; 7 Gastroenterology and Liver Diseases Research Center, Research Institute for Gastroenterology and Liver Diseases, Shahid Beheshti University of Medical Sciences, Tehran, 19835-178 Iran; 8 Department of Microbiology, School of Medicine, Tehran University of Medical Sciences, Tehran, 19835-178 Iran; 9 Thoracic Research Center, Imam Khomeini Hospital Complex, Tehran University of medical Sciences, Tehran, Iran

**Correction to: Rezasoltani et al. Gut Pathogens 2024 Feb 20;16(1):9**

10.1186/s13099-024-00604-0

Following publication of the original article [1], it was pointed out that the affiliation details for all authors were incorrectly processed in the authorship line and published incorrectly.

The original article has been updated.

The affiliation information was mistakenly published as: Sama Rezasoltani^1,7,8^, Mehdi Azizmohammad Looha^2,7^, Hamid Asadzadeh Aghdaei^2,7^, Seyedesomayeh Jasemi^3,7^, Leonardo Antonio Sechi^3,7,9*^, Maria Gazouli^4,7^, Amir Sadeghi^5,7^, Shirin Torkashvand^2,7^, Reyhaneh Baniali^2,7^, Hartmut Schlüter^1,7^, Mohammad Reza Zali^5,7^ and Mohammad Mehdi Feizabadi^3,6,7 *^

The affiliation information should read as: Sama Rezasoltani^1, 2^, Mehdi Azizmohammad Looha^3^, Hamid Asadzadeh Aghdaei^3^, Seyedesomayeh Jasemi^4^, Leonardo Antonio Sechi^4,5*^, Maria Gazouli^6^, Amir Sadeghi^7^, Shirin Torkashvand^3^, Reyhaneh Baniali^3^, Hartmut Schlüter^1^, Mohammad Reza Zali^7^, Mohammad Mehdi Feizabadi ^8,9*^

